# Trends in diagnostic methods and treatment of latent tuberculosis infection in a tertiary care center from 2000 to 2017

**DOI:** 10.1007/s10096-020-03850-7

**Published:** 2020-02-20

**Authors:** Jonathan W. Uzorka, Dinah L. Duinkerk, Lucia J. M. Kroft, Jaap A. Bakker, Rajen S. R. S. Ramai, Tom H. M. Ottenhoff, Sandra M. Arend

**Affiliations:** 1 Department of Infectious Diseases, Leiden University Medical Center, Albinusdreef 2, 2333 ZA Leiden, The Netherlands; 2 Faculty of Medicine, Leiden University, Albinusdreef 2, 2333 ZA Leiden, The Netherlands; 3 Department of Radiology, Leiden University Medical Center, Albinusdreef 2, 2333 ZA Leiden, The Netherlands; 4 Department of Clinical Chemistry and Laboratory Medicine, Leiden University Medical Center, Albinusdreef 2, 2333 ZA Leiden, The Netherlands; 5 Department of Pulmonology, Leiden University Medical Center, Albinusdreef 2, 2333 ZA Leiden, The Netherlands

**Keywords:** Latent tuberculosis, Tumor necrosis factor-alpha, Interferon-gamma release tests, Tuberculin test

## Abstract

Screening for latent tuberculosis infection (LTBI) is indicated before immunosuppressive therapies but is complicated by lack of a gold standard and limited by, e.g., immunosuppression. This study aimed to investigate a series of patients diagnosed with LTBI during screening before immunosuppressive therapy, describing how the use of diagnostic tests and treatment evolved over time. This retrospective cohort study included all individuals diagnosed with LTBI during screening before intended immunosuppressive therapy in a tertiary care hospital between January 2000 and December 2017. Evidence for LTBI, including history, tuberculin skin test (TST), QuantiFERON (QFT) result and suggestive lesions on chest radiography (CXR), and CT scan if available, was analyzed. The study included 295 individuals with LTBI, with median follow-up of 3.8 years (IQR 1.7–7.4 years). During screening, TST, QFT, and CXR were positive in 80.8%, 53.4%, and 22.7%, respectively. Chest CT revealed lesions associated with past tuberculosis infection in around 70%, significantly more frequent than CXR. In patients diagnosed with LTBI, we observed that the use of TST declined over time whereas the use of QFT increased, and that isoniazid was replaced with rifampicin as preferential treatment. Preventive treatment was started in 82.3%, of whom 88.6% completed treatment. During follow-up, no individuals developed active tuberculosis. The diagnosis of LTBI was based on history, TST, QFT, and/or CXR in nearly every possible combination, but mostly on TST and QFT. The most striking trends were the decreased use of TST, increased use of QFT, and the replacement of isoniazid with rifampicin for treatment.

**Electronic supplementary material:**

The online version of this article (10.1007/s10096-020-03850-7) contains supplementary material, which is available to authorized users.

## Introduction

Worldwide, nearly a quarter of all individuals is estimated to be latently infected with *Mycobacterium tuberculosis (Mtb)* [1]. Such latent tuberculosis infection (LTBI) has been defined as a persistent immune response to *Mtb*-specific antigens in the absence of evidence of active tuberculosis [2]. In individuals with LTBI, the lifetime risk of developing active tuberculosis is estimated at five to ten percent [3]. This risk is highest in infants, in recently infected individuals, in highly exposed individuals [4], and in persons with an impaired cellular immune response [5]. In individuals at high risk of progression to active disease, screening for LTBI and preventive treatment of those infected are recommended in order to reduce the risk of tuberculosis reactivation [6].

Screening for LTBI occurs by evaluating possible exposure to *Mtb* and by immunological testing and usually a chest radiograph (CXR) in order to exclude active disease or to reveal signs of old tuberculosis infection. Currently, two types of immunological tests are available to diagnose LTBI: the tuberculin skin test (TST) and interferon-gamma release assays (IGRAs). Test specificity of IGRAs is nearly 100%, which is substantially higher than that of the TST [7]. Test sensitivity for recent infection, for example during screening of tuberculosis contacts, is generally high although it varies depending on the study setting [8]. However, in patients with infection which was not recently acquired and/or in patients with an impaired cellular immune function such as patients treated with immunosuppressive drugs, sensitivity of TST and IGRA is lower [9, 10]. Immunocompromised patients, in whom the accuracy of TST and IGRA is limited, are often encountered in the setting of hospital care.

The primary aim of this study was to evaluate the contribution of different diagnostic methods to the clinical diagnosis of LTBI during screening of patients eligible for immunosuppressive therapy. Secondary aims were to evaluate how diagnostic methods and treatment of LTBI have evolved over time.

## Methods

### Study population and study design

The protocol of this retrospective observational study using anonymized data was approved by the Medical Ethics Committee (protocol G18.008) and waived from the requirement of patient informed consent. Eligible for inclusion were all individuals diagnosed with LTBI during screening prior to initiating immunosuppressive therapy or transplantation between January 2000 and December 2017 at Leiden University Medical Center (LUMC). Individuals with LTBI were identified by an automated search in all clinical correspondence from all screening departments (dermatology, emergency, endocrinology, gastro-enterology, hematology, infectious diseases, internal medicine, medical oncology, nephrology, ophthalmology, pulmonology, rheumatology, and transplantation) for the words (“latent*” OR “slapende,” being Dutch for dormant) AND (“tbc” OR “tuberculos*”). Exclusion criteria were active tuberculosis at the time of screening, treatment for active or latent tuberculosis in the past, diagnosis of LTBI before 2000, age < 18 years at the time of screening, or if no data with regard to the diagnosis of LTBI could be retrieved. STROBE guidelines for reporting cohort studies were followed [11].

### Data retrieval

Data were retrieved between March and September 2018 by DD and JU. Data from individuals diagnosed with LTBI prior to the introduction of the electronic patient record were obtained from archived paper files. If bacillus Calmette-Guerin (BCG) vaccination status had not been recorded, the “BCG World Atlas” interactive website [12] was consulted and vaccination status was assumed to be in accordance with the global BCG vaccination policies at the time of investigation. Follow-up data included any treatment for LTBI, evaluation of whether immunosuppressive therapy was started or changed, and development of active tuberculosis during follow-up. Patients were considered lost to follow-up if they had not visited the hospital within one year following LTBI diagnosis. Immunosuppression was both qualitatively and quantitatively assessed. For the quantitative analysis, a drug score as designed by Sester et al. [13] was used with the following additions: leflunomide and methotrexate were scored as 0.7, any form of chemotherapy as 1.0, and TNF-antagonist therapy as 1.3 (Supplementary Table S1).

### TST and QFT

A TST result ≥ 10 mm of induration was considered positive (see Supplementary Methods for additional specifications). All IGRA performed at the LUMC consisted of the QuantiFERON (QFT). For this study, the time of interest was divided in four periods as based on the use of different IGRA formats. Until 2004, QFT was only used in research setting. From 2004 to 2007, a commercially available second generation QFT was used (liquid antigen added to whole blood in culture plates). In 2007, this was replaced by the QFT-Gold-in Tube and since August 2016, QFT-Gold Plus was used. An unquantified negative or positive QFT result was regarded to be 0.00 IU/ml or 1.00 IU/ml, respectively.

### Radiology

Radiographic data were obtained from the original reports of radiologists. Radiographic tests used were CXRs and CT scans. All findings on the characteristics of past tuberculosis or LTBI listed in the original report were recorded, i.e., (non)-calcified nodules, fibrotic scarring, and pleural thickening that may have been a result of tuberculosis infection in the past, further referred to as CXR or CT positive. A CT was in most cases performed as part of routine practice and not specifically aimed at lesions due to past tuberculosis infection. Lesions on CT scan were only compared with those found on CXR if the time interval between a CT scan and CXR was < 12 months.

### Statistical analyses

Statistical analyses were performed using IBM SPSS Statistics 25 and Graph Pad Prism version 8.0. Categorical data were assessed using the chi square test or Fishers’ exact test where appropriate. Continuous data were compared using the One-way ANOVA test or Mann-Whitney U and Kruskal-Wallis in case of a non-parametric distribution of data. We assessed the level of agreement between diagnostic tests with Kappa statistics. *P* values of < 0.05 were considered statistically significant. Multiple regression analyses were conducted to examine the effect of age, immigration, BCG vaccination status, exposure, immune mediated inflammatory disease (IMIDs), and immune status on QFT, TST, and CXR results.

## Results

### Patient characteristics

**Fig. 1:**
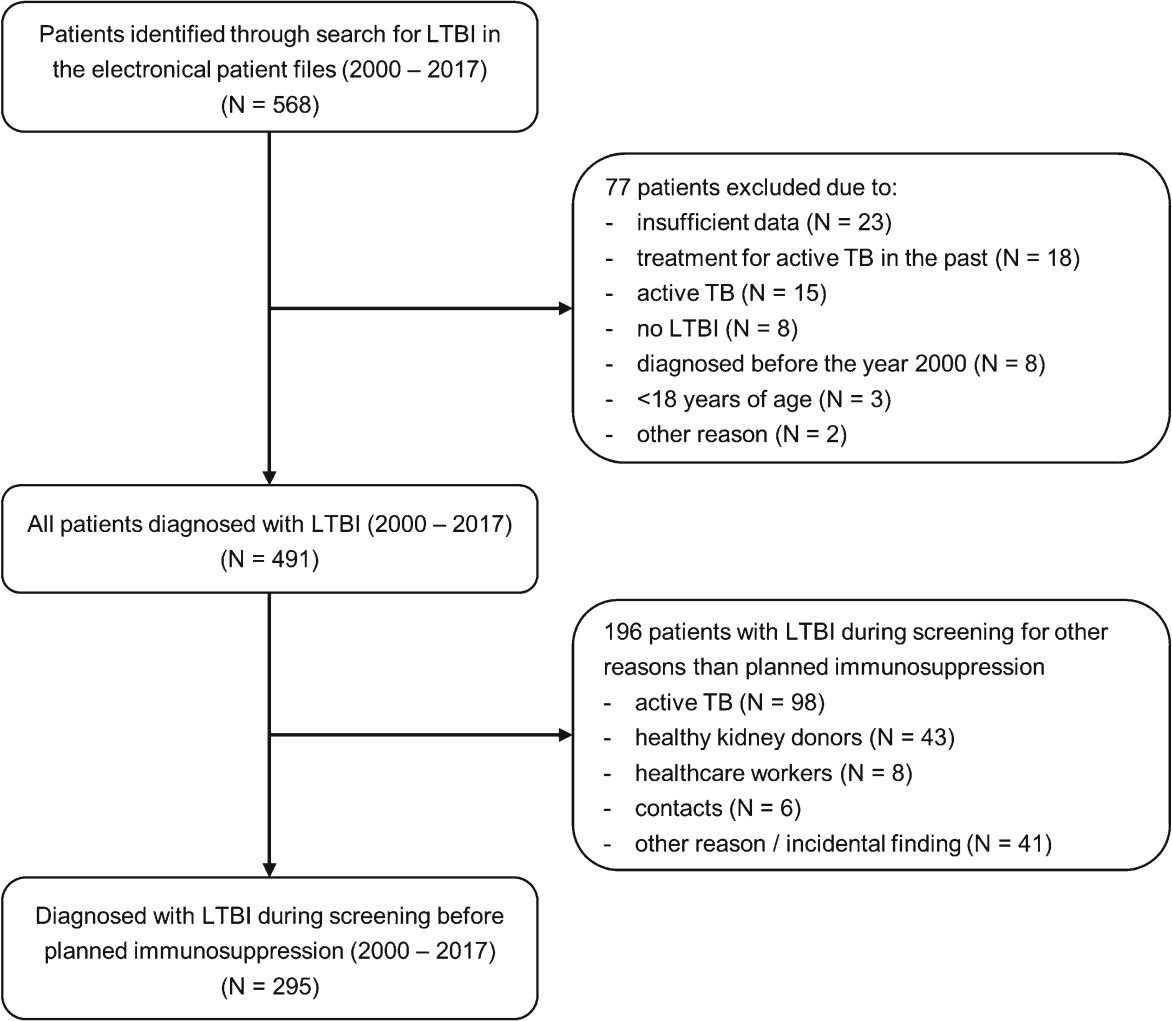
Flow diagram. LTBI, latent tuberculosis infection; TB, tuberculosis

**Fig. 2:**
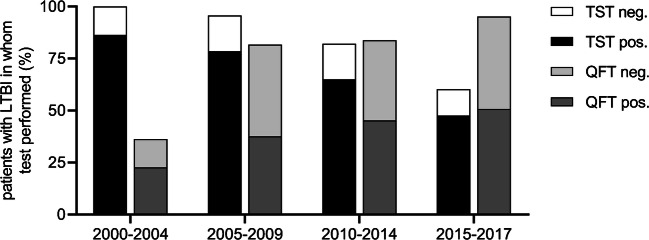
Trends in the proportion of individuals in whom a TST or QFT was performed (including test results). QFT, QuantiFERON-TB Gold In-Tube/ Plus; TST, tuberculin skin test

The search identified 568 individuals with LTBI, of whom 77 were excluded as shown in Fig. 1. Of the remaining 491 individuals, 295 (60.1%) were diagnosed with LTBI during screening prior to initiation of immunosuppressive therapy and included for analysis. Patients were mainly screened at the departments of rheumatology (*N* = 132), gastro-enterology (*N* = 71), and nephrology (*N* = 42). Median follow-up time was 3.8 years (IQR 1.7–7.4 years). There were no significant differences with regard to socio-demographic characteristics between the different time periods (Table 1). The proportion of BCG-vaccinated individuals remained similar over time. Overall, the number of patients tested by TST and/or QFT was similar (245/295 versus 242/295, respectively). However, the percentage of individuals in whom a TST had been performed decreased over time from 100 to 60.3%, whereas the percentage tested by QFT increased from 36.4 to 95.2% (Fig. 2).

**Table 1:** Characteristics of all 295 persons diagnosed with LTBI during screening from 2000 to 2017

	Period of LTBI screening					
	(2000–2004)	(2005–2009)	(2010–2014)	(2015–2017)	All	
Characteristic	*N* = 22	*N* = 93	*N* = 117	*N* = 63	*N* = 295	*p* value
Age (year)	53.6 ± 11.8	54.9 ± 11.9	54.5 ± 14.5	56.6 ± 15.0	55.0 ± 13.6	0.74
Sex (male)	17/22 (77.3)	51/93 (54.8)	69/117 (59.0)	37/63 (58.7)	174/295 (59.0)	0.30
Immigrant	12/22 (54.5)	36/93 (38.7)	62/117 (53.0)	36/63 (57.1)	146/295 (49.5)	0.09
TB history	1/22 (4.5)	17/93 (18.3)	26/117 (22.2)	12/63 (19.0)	56/295 (19.0)	0.28
Active TB, not treated	0/22 (0)	3/93 (3.2)	4/117 (3.4)	1/63 (1.6)	8/295 (2.7)	0.90
Latent TB, not treated	1/22 (4.5)	14/93 (15.1)	22/117 (18.8)	11/63 (17.5)	48/295 (16.3)	0.42
Travel to TB-endemic country^a^	8/22 (36.4)	29/93 (31.2)	51/117 (43.6)	29/63 (46.0)	117/295 (39.7)	0.19
TB contact	9/22 (40.9)	32/93 (34.4)	15/117 (12.8)	11/63 (17.5)	67/295 (22.7)	< 0.001
Risk group (any)	3/22 (13.6)	22/93 (23.7)	24/117 (20.5)	20/63 (31.7)	69/295 (23.4)	0.24
Alcohol abuse	3/22 (13.6)	4/93 (4.3)	9/117 (7.7)	3/63 (4.8)	19/295 (6.4)	0.34
Drug abuse	1/22 (4.5)	0/93 (0)	1/117 (0.9)	0/63 (0)	2/295 (0.7)	0.190
Homeless	0/22 (0)	0/93 (0)	0/117 (0)	0/63 (0)	0/295 (0)	
Prison inmate	0/22 (0)	0/93 (0)	1/117 (0.9)	5/63 (7.9)	6/295 (2.0)	0.009
Occupational^b^	0/0 (0)	19/93 (20.4)	15/117 (12.8)	14/63 (22.2)	48/295 (16.3)	0.03
Comorbidities (any)	21/22 (95.5)	88/93 (94.6)	112/117 (95.7)	59/63 (93.7)	280/295 (94.9)	0.94
Auto-immune disease	8/22 (36.4)	72/93 (77.4)	55/117 (47.0)	31/63 (49.2)	166/295 (56.3)	< 0.001
Chronic lung disease	0/22 (0)	3/93 (3.2)	4/117 (3.4)	7/63 (11.1)	14/295 (4.7)	0.11
Diabetes mellitus (type-2)	4/22 (18.2)	7/93 (7.5)	17/117 (14.5)	8/63 (12.7)	36/295 (12.0)	0.31
HIV	0/22 (0)	0/93 (0)	0/117 (0)	0/63 (0)	0/295 (0)	
Liver cirrhosis	10/22 (45.5)	12/93 (12.9)	24/117 (20.5)	7/63 (11.1)	53/295 (18.0)	0.003
Malignancy	2/22 (9.1)	4/93 (4.3)	11/117 (9.4)	6/63 (9.5)	23/295 (9.5)	0.44
Dialysis/chronic kidney failure	1/22 (4.5)	4/93 (4.3)	29/117 (24.8)	19/63 (30.2)	53/295 (18.0)	< 0.001
Immunocompromised (at screening)^c^	7/22 (31.8)	50/93 (53.8)	40/117 (34.2)	26/63 (41.3)	123/295 (41.7)	0.031
BCG vaccinated	9/22 (40.9)	48/93 (51.6)	61/117 (52.1)	27/63 (42.9)	145/295 (49.2)	0.52
TST performed at time of screening	22/22 (100)	89/93 (95.7)	96/117 (82.1)	38/63 (60.3)	245/295 (83.1)	< 0.001
QFT performed at time of screening	8/22 (36.4)	76/93 (81.7)	98/117 (83.8)	60/63 (95.2)	242/295 (82.0)	< 0.001
Radiology						
Chest X-ray available	21/22 (95.5)	87/93 (93.5)	108/117 (92.3)	57/63 (90.5)	273/295 (92.5)	0.88
CT scan available	5/22 (22.7)	13/93 (14.0)	41/117 (35.0)	17/63 (27.0)	76/295 (25.8)	0.006

Continuous variables are displayed as mean ± SD, categorical values are displayed as numerator over denominator (%)

*LTBI*, latent tuberculosis; *TB*, tuberculosis; *BCG*, bacillus Calmette-Guérin; *TST*, tuberculin skin test; *QFT*, QuantiFERON-TB Gold In-Tube/Plus

^a^Defined as country with yearly tuberculosis incidence ≥ 40 cases of active tuberculosis/100,000 inhabitants

^b^Healthcare worker, employed at a refugee/asylum center etc.

^c^Immunosuppression received within 2 months prior to screening

### Contribution of different indicators of LTBI

**Fig. 3:**
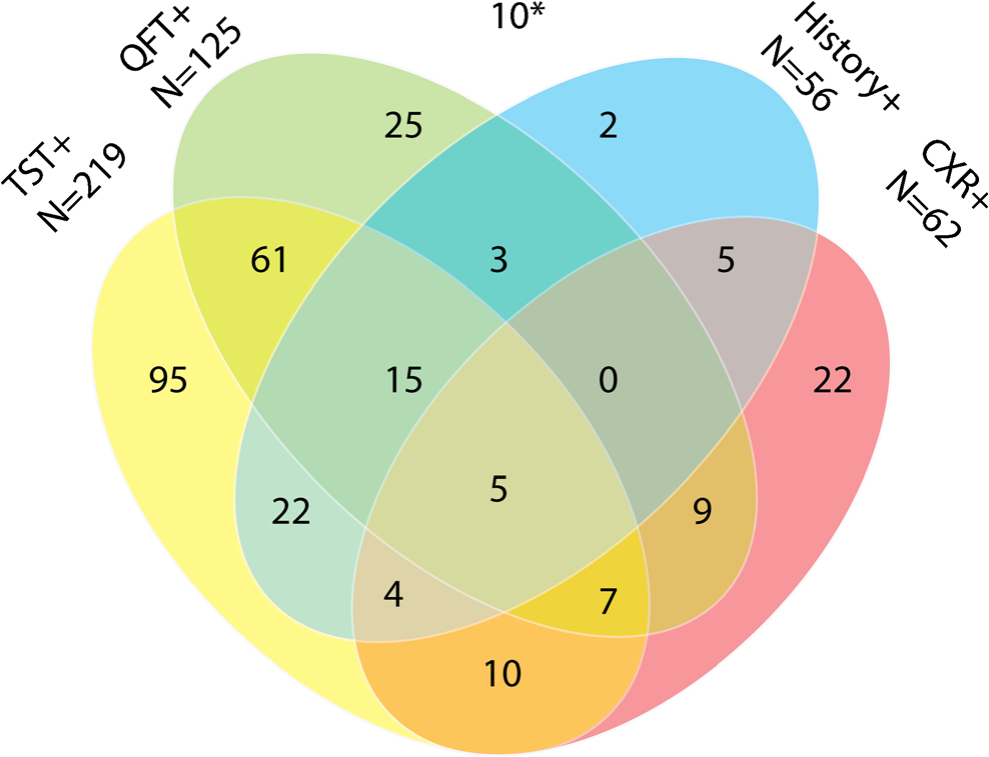
Venn diagram of contribution of positive TST, QFT, history, and CXR results to the diagnosis of LTBI during screening. A structured overview showing which (combination of) diagnostic methods contributed to the diagnosis of LTBI. TST+, QFT+, history+, and CXR+ were defined as a TST induration ≥ 10 mm, QFT result ≥ 0.35 IU/mL, history of (latent) tuberculosis, and lesions on CXR suggestive for a prior tuberculosis infection, respectively. TST, tuberculin skin test; QFT, QuantiFERON-TB Gold In-Tube/Plus; CXR, chest radiography; *of 10 patients with a diagnosis of LTBI despite negative TST, QFT, X, and history of (latent) tuberculosis, four individuals were diagnosed with LTBI due to tuberculosis-related lesions on a CT scan, of whom one also reported being exposed to active tuberculosis. In three patients, all of whom had renal failure, diagnosis was based on a TST induration size ranging from 5 to 9 mm. In one case, diagnosis was based only on anamnestic exposure to active tuberculosis; this patient was also immunosuppressed during screening. In two patients, the reasons for LTBI diagnosis were unclear

Positive findings for LTBI varied between individuals. A positive TST was the most frequent finding (*N* = 219) (Fig. 3), while a history of untreated LTBI or active tuberculosis was found least often (*N* = 56). In 38 patients, LTBI was diagnosed without a positive TST or IGRA result, of whom seven had a positive history for tuberculosis or LTBI and 27 had suggestive lesions on CXR as the only indication of LTBI.

### TST and QFT

TST results were available in 245 individuals, valid QFT results in 234, after exclusion of eight individuals with an indeterminate QFT result. The effect of clinical characteristics on the QFT and TST results is shown in Table 2. The overall frequency of positive test results was significantly higher for TST (198/245; 80.8%) than for QFT (125/234; 53.4%, *p* < 0.001), but in the last time period, the absolute number of positive QFT results just exceeded the number of positive TST results (Fig. 2). IMIDs or immunosuppressive therapy were significantly associated with a negative TST or QFT result.

**Table 2:** Comparison between individuals with a positive or negative test result

	TST < 10 mm	TST ≥ 10 mm	All		QFT−	QFT+	All	
Characteristic	*N* = 47	*N* = 198	*N* = 245	*p* value	*N* = 109	*N* = 125	*N* = 234	*p* value
Age (year)	60.0 ± 14.6	52.4 ± 12.5	53.9 ± 13.2	< 0.001	56.4 ± 13.2	54.4 ± 14.3	55.3 ± 13.8	0.27
Sex (male)	26/47 (55.3)	120/198 (60.6)	146/245 (59.6)	0.51	58/109 (53.2)	75/125 (60.0)	133/234 (56.8)	0.30
Immigrant	12/47 (25.5)	110/198 (55.6)	122/245 (49.8)	< 0.001	35/109 (32.1)	73/125 (58.4)	108/234 (46.2)	< 0.001
High TB-endemic country	6/47 (12.8)	55/198 (27.8)	61/245 (24.9)	0.03	17/109 (15.6)	45/125 (34.2)	62/234 (26.5)	0.001
History of TB or LTBI	7/47 (14.9)	26/198 (13.1)	33/245 (13.5)	0.75	23/109 (21.1)	23/125 (18.4)	46/234 (19.7)	0.60
Exposure	15/47 (31.9)	41/198 (20.7)	56/245 (22.9)	0.10	28/109 (25.7)	28/125 (22.4)	56/234 (23.9)	0.56
IMID	34/47 (72.3)	109/198 (55.1)	143/245 (58.4)	0.03	83/109 (76.1)	65/125 (52.0)	148/234 (63.2)	< 0.001
Renal failure/dialysis	5/47 (10.6)	42/198 (21.2)	47/245 (19.2)	0.10	14/109 (12.8)	21/125 (16.8)	35/234 (15.0)	0.40
Immunosuppressed	27/47 (57.4)	75/198 (37.9)	102/245 (41.6)	0.01	59/109 (54.1)	46/125 (36.8)	105/234 (44.9)	0.008
Drug score^a^	0.65 ± 0.7	0.40 ± 0.6	0.45 ± 0.6	0.01	0.55 ± 0.6	0.41 ± 0.6	0.47 ± 0.6	0.08
BCG vaccination	13/47 (27.7)	109/198 (55.1)	122/245 (49.8)	0.001	45/109 (41.3)	65/125 (52.0)	110//234 (47.0)	0.10
TST positivity					69/95 (72.6)	77/92 (83.7)	146/187 (78.1)	0.070
TST induration (mm)					11.7 ± 8.1	15.4 ± 9.6	13.5 ± 9.0	0.004
QFT positivity^b^	15/41 (36.6)	77/146 (52.7)	92/187 (49.2)	0.08				
CXR-lesions^c^	26/45 (57.8)	19/184 (10.3)	45/229 (19.7)	< 0.001	29/102 (28.4)	21/115 (18.3)	50/209 (23.0)	0.08
CT-lesions^d^	15/16 (93.8)	22/35 (62.9)	37/51 (72.5)	0.04	19/22 (86.4)	25/40 (62.5)	44/62 (71.0)	0.05

Continuous variables are displayed as mean ± SD, categorical values as numerator over denominator with characteristic available (%)

*QFT*, QuantiFERON-TB Gold In-Tube/Plus; *TST*, tuberculin skin test; *TB*, tuberculosis; *LTBI*, latent tuberculosis infection; *IMID*, immune mediated inflammatory disease; *BCG*, bacillus Calmette-Guérin; *CXR*, chest radiography

^a^Defined in the methods section

^b^Indeterminate QuantiFERON results were excluded from this analysis

^c^Lesions suggestive of prior tuberculosis infection on chest radiography

^d^Lesions suggestive of prior tuberculosis infection on computed tomography

In 187 individuals, both TST and QFT were available. The agreement between both tests was poor (κ = 0.11; 95% CI, 0.05–0.17). Test results were concordant positive in 77/187 (41.2%), concordant negative in 26/187 (13.9%), discordant TST+/QFT− 69/187 (36.9%) and TST−/QFT+ in 15/187 (8.0%) individuals. Of patients with a concordant positive, discordant TST and QFT or concordant negative results, around one third, half and two third, respectively, were immunosuppressed at the time of screening (data not shown). Twelve individuals had a TST induration between 5 and 9 mm, of whom eight had a positive QFT result, while seven patients had a QFT result in the borderline range (0.15–0.35 IU/ml), of whom six had a positive TST result (Table 3). BCG-vaccinated individuals with a positive TST slightly more often had a positive QFT result compared to BCG-unvaccinated individuals with a positive TST result (57.7% versus 47.1%), irrespective of immune status (Supplementary Table S2). Of note, no significant differences were found in this regard when QFT-Plus was compared with QFT-Gold-in Tube. The impact of patient characteristics on TST/QFT was further investigated in a regression analysis (Supplementary Table S3).

**Table 3:** QuantiFERON results by tuberculin skin test result category

	Tuberculin skin test result			
	< 5 mm	5–9 mm	≥ 10 mm	All
QuantiFERON result	*N* = 29	*N* = 12	*N* = 146	*N* = 187
< 0.15 IU/mL	21 (72.4)	4 (33.3)	63 (43.2)	88 (47.1)
0.15–0.35 IU/mL	1 (3.4)	0 (0)	6 (4.1)	7 (3.7)
≥ 0.35 IU/mL	7 (24.1)	8 (66.7)	77 (52.7)	92 (49.2)

Values are displayed as numerator over denominator (%)

### Radiography

CXR showed at least one finding suggestive of prior *Mtb*-infection in 62/273 (22.7%) individuals with a CXR available (Table 4). A calcified nodule was most frequently observed (*N* = 19). A CT scan was available in 76 (25.8%) and revealed at least one lesion considered suggestive of past tuberculosis in 53/76 (69.7%), most often a calcified nodule. In 59 individuals, both CXR and a CT scan were available (Table 5). Of those, a CT scan showed significantly more often lesions suggestive of past tuberculosis infection (38/59; 64.4%) compared with CXR (20/59; 33.9%, *p* = 0.018). Concordance between CXR and CT scan for revealing such lesions was low (59.3%; Cohen’s κ = 0.26). The impact of patient characteristics on abnormalities on CXR was assessed (Supplementary Table S3). Only a higher age was associated with lesions suggestive of LTBI on CXR (OR 1.04; 95% CI, 1.02–1.06, per year increase in age).

**Table 4:** Proportion of lesions consistent with prior tuberculosis infection identified by chest radiography or CT scan

	CXR	CT	
Characteristic	*N* = 273	*N* = 76	*p* value
Lesions on chest imaging^a^	62/273 (22.7)	53/76 (69.7)	< 0.001
Calcified nodule	19/273 (7.0)	32/76 (42.1)	< 0.001
Non-calcified nodule	17/273 (6.2)	22/76 (28.9)	< 0.001
Fibrotic scarring	9/273 (3.3)	8/76 (10.5)	0.007
Pleural thickening	13/273 (4.8)	7/76 (9.2)	0.22
Other	11/273 (4.0)	3/76 (3.9)	1.00

Values are displayed as numerator over denominator (%)

*CXR*, chest radiography; *CT*, computed tomography

^a^Lesions suggestive of prior tuberculosis infection on chest imaging

**Table 5:** Agreement between chest radiography and a CT scan regarding lesions consistent with prior tuberculosis on imaging

	CXR-lesions^a^		
	Negative	Positive	All
CT-lesions^b^	*N* = 39	*N* = 20	*N* = 59
Negative	18 (46.2)	3 (15.0)	21 (35.6)
Positive	21 (53.8)	17 (85.0)	38 (64.4)

Values are displayed as numerator over denominator (%). Only individuals in whom the time interval between a CT scan and CXR was < 12 months were included in this analysis

*CXR*, chest radiography; *CT*, computed tomography

^a^Lesions suggestive of prior tuberculosis infection on chest radiography

^b^Lesions suggestive of prior tuberculosis infection on computed tomography

### Treatment and follow-up

**Fig. 4:**
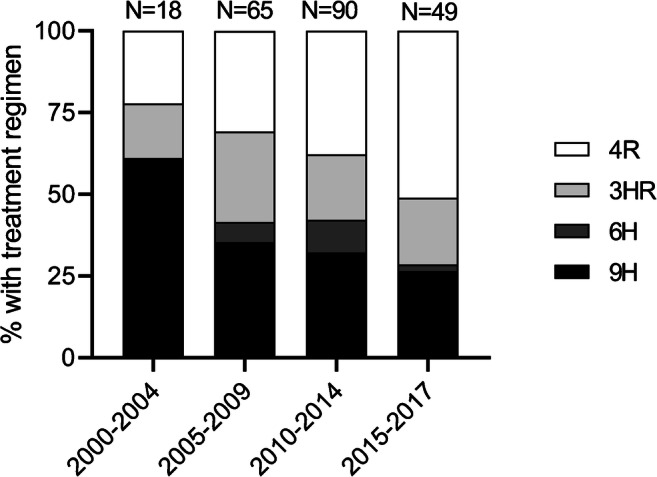
Trends in the treatment regimen among individuals with LTBI who started anti-tuberculous therapy. 4R, 4 months rifampicin; 3HR, 3 months isoniazid and rifampicin; 6H, 6 months isoniazid; 9H, 9 months isoniazid

Supplementary Table S4 shows the follow-up data. Preventive treatment was started in 241/293 (82.3%) individuals with LTBI. Of patients treated with isoniazid and/or rifampicin, 192/214 (89.7%) completed the treatment (Supplementary Table S5). The most striking trend with regard to treatment regimens was the decrease of the use of 6 months isoniazid in favor of a shorter regimen with rifampicin, as depicted in Fig. 4. The proportion of individuals who experienced adverse events during treatment was 101/212 (47.6%). Immunosuppressive therapy was continued unchanged in 20/291 cases (6.9%) and was intensified or newly started in 202/291 (69.4%), see Supplementary Table S6. Twenty patients started immunosuppressive therapy without receiving preventive treatment for varying reasons as explained in Supplementary Table S6. Only two patients who refused LTBI treatment later received high dose steroids or a TNF antagonist. None of the included individuals had developed active tuberculosis during follow-up.

## Discussion

This study aimed to evaluate which diagnostic methods had been used in patients diagnosed with LTBI during screening before intended immunosuppressive therapy. In patients diagnosed with LTBI, we observed that the TST became relatively less frequently used over the years with a concomitant increase of the use of QFT, but that a positive TST remained the most frequent positive finding over time. Radiological lesions associated with LTBI or past tuberculosis were significantly more often seen on a CT scan than on CXR.

### Screening for LTBI

While it is generally agreed that screening for LTBI is indicated before immunosuppressive treatment, there is less agreement on how such screening should be done. A recent systematic review of 38 guidelines compared recommendations for diagnosing LTBI in (future) immunocompromised patients [14]. A TST, QFT, CXR, or taking into account an anamnestic history of previous tuberculosis infection was recommended by 34/38 (89.5%), 26/38 (68.4%), 18/38 (47.4%), and 21/38 (55.3%) guidelines, respectively, highlighting the lack of concordance. Most guidelines recommended using more than one screening method, but only four recommended taking into account all possible evidence during screening for LTBI.

### Contribution of TST and QFT

We assumed that all positive findings had been used to diagnose LTBI, as illustrated in Fig. 3. As expected, LTBI was mainly diagnosed based on a positive TST or QFT result, with a positive TST being most frequent. The proportion of positive QFT results was lower than the proportion of positive TST results and there were more cases with discordant TST+/QFT− than TST−/QFT+ results. QFT was used in a larger proportion of patients over time while the proportion of individuals with LTBI in whom a TST had been performed decreased. The reasons for this trend could not be studied, but it is possible that some physicians regarded the QFT as a convenient alternative for the TST because of logistical advantages. In patients in whom both TST and QFT were available, a borderline QFT result was often associated with a positive TST. This confirms previous studies which showed that borderline QFT reflect LTBI in the majority of cases [15, 16]. In patients at high risk of reactivation following immunosuppression, such borderline results during screening are clinically relevant [17].

### Contribution of radiology

CXR contributed to the diagnosis of LTBI, but substantially less compared with TST and QFT. This finding reflects the low sensitivity of the CXR for LTBI, which is around 15% as estimated by a recent systematic review [18]. A CT scan was significantly more sensitive (69.7%) for lesions associated with past tuberculosis infection compared with CXR, which was consistent with previous findings [19–21]. A previous study that reported on old tuberculosis-related lesions on a CT scan in pre-transplant patients with a positive TST found a similar proportion (71.4%) [22], while it was lower (44%) in another study, conducted in individuals with a positive T-SPOT result [23]. The benefits of a CT scan during screening for LTBI would consist of its high sensitivity for tuberculosis-related lesions and independence of an individual’s immune status. However, the specificity of certain findings supposedly suggestive for old tuberculosis infection on a CT scan has not yet been determined, and a CT scan is limited by its high costs and radiation dose. It would be useful, however, to reassess a prior CT scan made for other purposes for lesions suspect of prior tuberculosis.

### Limitations

Our observational retrospective study was subject to potential information bias, but we expect that this form of bias was similar across the different time periods and analyzed groups. Secondly, our approach to study patients diagnosed with LTBI and to assess diagnostic methods used to reach this diagnosis relied on interpretation of test results by the treating physician. Ideally, screening is always performed before any immunosuppressive therapy is started but in practice this is not always done and some patients in our study already used immunosuppression at that time of screening. However, our approach provided useful insight in the real-life diagnostic process in clinical practice.

### Conclusions

Overall, a positive TST contributed most frequently to the diagnosis, indicating that this old test retains its diagnostic value. Given the current lack of a gold standard for LTBI and the frequent occurrence of discordant LTBI diagnostic test results, an inclusive approach including all available evidence for a tuberculosis infection seems the most effective strategy in screening patients prior to initiation of immunosuppressive therapy. Our study also suggests that the use of a chest CT scan could have additional value in diagnosing LTBI.

## Electronic supplementary material

ESM 1 (DOCX 86 kb)
