# Supplementary material for: Trends in diagnostic methods and treatment of latent tuberculosis infection in a tertiary care center from 2000 to 2017
Source: Eur J Clin Microbiol Infect Dis. 2020 Feb 20;39(7):1329–37. doi: 10.1007/s10096-020-03850-7 (PMC7303080; doi:10.1007/s10096-020-03850-7)
Supplement: Supplementary file 1 — (DOCX 86 kb) [file 10096_2020_3850_MOESM1_ESM.docx]

**Supplementary Materials**

**Supplementary Methods**

*Tuberculin skin test (TST)*

If TST results were only recorded as negative or positive these were scored as zero or 10 mm, respectively, except if there was any mention of rash or blisters, in which case the TST was arbitrarily assigned an induration of 25 mm. In cases where TST was performed during childhood or youth, and the patient could not provide a specific year, age was assumed to be 10 or 15 years, respectively.

**Supplementary tables**

**Table S1. Cumulative drug score**

Drug score as developed and supplemented by:

Sester M, van Leth F, Bruchfeld J, Bumbacea D, Cirillo DM, Dilektasli AG, et al. Risk assessment of tuberculosis in immunocompromised patients. A TBNET study. Am J Respir Crit Care Med. 2014;190(10):1168-76.

**Table S2. Test results by BCG vaccination**

Abbreviations: BCG: Bacillus Calmette-Guérin, TST: tuberculin skin test; QFT: QuantiFERON.

**Table S3. Regression analysis of factors associated with test positivity for latent tuberculosis infection**

Abbreviations: TST: tuberculin skin test; QFT: QuantiFERON; CXR: chest radiography; IMID: immune modifying inflammatory disease; BCG: Bacillus Calmette-Guérin.

^a^ TST was considered positive if induration size was ≥ 10 mm

^b^ QFT was considered positive according to manufacturer cut-off, being 0.35 IU/ml

^c^ CXR was considered postive in case at least one lesion on CXR indicative for prior tuberculosis or latent tuberculosis infection was identified.

**Table S4. Follow-up and evolution of preventive treatment in 295 persons diagnosed with LTBI from 2000 - 2017**

Categorical values are displayed as numerator over denominator (%).

Abbreviations: LTBI: latent tuberculosis infection; 6H: six months isoniazid; 9H: nine months isoniazid; 4R: four months rifampicin; 3HR: three months combination therapy of isoniazid and rifampicin. ULN: upper limit of normal; TB: tuberculosis

**Table S5. Characteristics of anti-tuberculous treatment by regimen**

Abbreviations: ULN: upper limit of normal.

**Table S6. Initiation of immunosuppressive therapy by anti-tuberculous treatment**

Abbreviations: IS: immunusuppresive therapy.

^a^ These 20 patients did not receive preventive treatment for LTBI because immunosuppresive therapy was low-dose and/or for a short period of time (N=7), contra-indicated or refused by patient (N=5), referred to lead physician elsewhere (N=2), other reasons (N=5), or had died (N=1).
